# Vascular pathology and pathogenesis of cognitive impairment and dementia in older adults

**DOI:** 10.1016/j.cccb.2022.100148

**Published:** 2022-06-30

**Authors:** Sonal Agrawal, Julie A. Schneider

**Affiliations:** a Rush Alzheimer's Disease Center, Rush University Medical Center, Jelke Building, 1750 W. Harrison Street, Chicago 60612, IL, USA; b Department of Pathology, Rush University Medical Center, Chicago, IL, USA

**Keywords:** Vascular cognitive impairment, Dementia, Vascular pathology, Infarcts, Alzheimer's disease, Mixed pathology

## Abstract

It is well recognized that brains of older people often harbor cerebrovascular disease pathology including vessel disease and vascular-related tissue injuries and that this is associated with vascular cognitive impairment and contributes to dementia. Here we review vascular pathologies, cognitive impairment, and dementia. We highlight the importance of mixed co-morbid AD/non-AD neurodegenerative and vascular pathology that has been collected in multiple clinical pathologic studies, especially in community-based studies. We also provide an update of vascular pathologies from the Rush Memory and Aging Project and Religious Orders Study cohorts with special emphasis on the differences across age in persons with and without dementia. Finally, we discuss neuropathological perspectives on the interpretation of clinical-pathological studies and emerging data in community-based studies.

## Highlights

• Cerebrovascular pathologies are common in the brains of older people and severity increase with advancing age.
• Vascular pathologies associate with the development of VCI and contribute to the clinical picture judged to be Alzheimer's disease.
• Vascular pathology often coexists along with other brain pathologies, most frequently with Alzheimer's disease, a phenomenon known as mixed pathologies.
• Mixed vascular and AD pathologies have increased role in the pathogenesis of cognitive impairment and development of dementia.

## 1 Introduction

The term vascular cognitive impairment (VCI) was introduced around the start of the new millennium from the concept of vascular dementia (VD). VD is the second most common cause of dementia after Alzheimer's disease, accounts for an estimated 15% of dementia cases in Europe [1], about 20% in North America [2], and with higher estimates of around 30% in Asia and developing countries [3], [4], [5]. VCI covers all forms of cognitive impairment associated with cerebrovascular diseases or with vascular risk factors from mild cognitive impairment to dementia syndrome [6], [7], [8]. The contribution of vascular pathologies and vascular-related risk factors to VCI have been widely reported in both population and autopsy-based studies [9], [10], [11], [12], [13], [14]. Vascular-related pathological changes are frequent with increased age, often coexist along with other brain pathologies, most frequently with Alzheimer's disease (AD) pathology and varying degrees with other neurodegenerative pathologies, a phenomenon known by various names including mixed pathologies or multiple pathologies [15], [16], [17].

In this review, we discuss vessel-related neuropathologies and tissue injury in autopsy-based studies and their association with the development of cognitive impairment and clinical picture of dementia judged to be Alzheimer's disease; followed by a summary of postmortem studies that have layed much of the groundwork in the field of mixed vascular and AD brain pathologies and detail on pathophysiologic mechanisms underlying the development of VCI, Alzheimer's and mixed dementias. Finally, we conclude with the current state of the literature and recommendations for future directions.

## 2 Common vessels disease causes vascular lesions in VCI and dementia

It is well recognized that cerebrovascular disease including atherosclerosis, arteriolosclerosis, cerebral amyloid angiopathy (CAA), infarcts, and other tissue injuries are associated with the development of VCI and contribute to the dementia that clinically judged to be Alzheimer's disease. These cerebrovascular diseases are frequently present in the brains of older people and frequency and severity increase with advancing age. Neuroimaging and pathological studies each identify some of these cerebrovascular diseases, however, the pathophysiological mechanism underlying VCI or dementia can be elusive, especially in the absence of large or multifocal strategic lesions. This may be related to numerous factors including the complexity of the cerebrovascular disease, coexisting with AD pathology, occurring commonly in older people with and without dementia, the heterogeneity of severity across locations, and the absence of associated clinical stroke. Less common/rare forms of vessels disease associated with VCI including genetic disorders, e.g., CADASIL, CARASIL, etc, will also be discussed.

### 2.1 Large vessel atherosclerosis

**Fig. 1:**
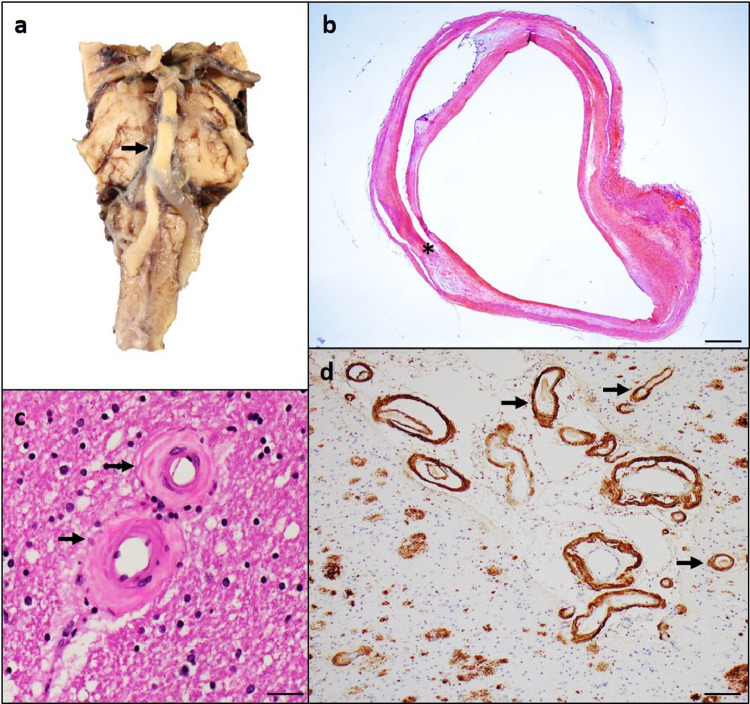
– Vessels disease associated with VCI and dementia The presence of yellow atherosclerotic plaque in the basilar artery of Circle of Willis can alter normal arteries into thickened and non-transparent tubes (arrow). Atheroma in the basilar artery invades onto the lumen causing narrowed lumen (a). Other common histologic features include fibromuscular intima hyperplasia with an undamaged endothelium and the presence of cholesterol clefts (b, asterisk). Arteriolosclerosis of the smaller penetrating arteries (c) and cerebral amyloid angiopathy in the meningeal and parenchymal vessels (arrows, d). Scale bars: 5mm (images b); 200µm (image d); 50µm (image c).

The term “atherosclerosis” is derived from Greek origin, meaning thickening of the intimal layer of arteries and accumulation of fat [18]. Atherosclerosis commonly occurs in the extracranial and intracranial arteries of the aging brain, most commonly the basilar and carotid arteries, and the circle of Willis. Pathogenic mechanisms involved in forming atherosclerotic plaques include intima proliferation, accumulation of lipid-laden macrophages, and buildup of cholesterol within the vessel wall (Fig. 1a and b). These processes result in the generation and calcification of atherosclerotic plaques, necrosis, and finally result in the narrowing of the arterial lumen, leading to compromised blood flow, subsequent potential vessel rupture, and local thrombosis. Many downstream pathological mechanisms are projected by which atherosclerotic cerebrovascular pathology could be associated with VCI or dementia including cerebral embolism originating from ruptured or thrombotic carotid plaques and targeting distal vessels, thrombotic occlusion of large vessels with subsequent chronic cerebral hypoperfusion, blood pressure dysfunction disturbing blood-brain barrier (BBB) integrity, increased parenchymal oxidative stress, and inflammation [6,[19], [20], [21], [22]]

A Prospective Cardiovascular Munster study identified 8 risk factors including age, LDL cholesterol, smoking, HDL cholesterol, systolic blood pressure, myocardial infarction, diabetes mellitus, and triglycerides related to the severity of atherosclerosis [23]. The black older population may have more severe atherosclerosis pathology than the white older population [24].

A large percentage of participants diagnosed with dementia (for example: more than 45% in one study examination (Arvanitakis et al., 2016a) and approximately 36% of in another study [25], had the presence of moderate-to-severe atherosclerosis in the circle of Willis observed on gross. Atherosclerosis can cause gross brain infarcts, whereas embolism of atherogenic thrombi can lead to a broad variety of infarcts and microinfarcts [26]. More recently, data from the Rush Religious Orders Study (ROS) and Memory and Aging Project (MAP) study show that moderate-to-severe atherosclerosis is related to the presence of multiple microinfarcts and separately with subcortical microinfarcts [27]. In addition, the ROS and MAP study indicated a positive association between atherosclerosis and brain structural alteration such as cortical atrophy and hippocampal atrophy [28].

### 2.2 Arteriolosclerosis

Brain arteriolosclerosis, a common subtype of small vessel disease, is characterized by pathologic arteriolar wall thickening, stenosis, and narrowing of the lumen of small arteries and arterioles 40 to--150 μm in diameter (Fig. 1c). Arteriolosclerosis has been documented as two distinct subtypes: hyaline type i.e., deposition of glassy-looking amorphous material in the arteriolar wall, and hyperplastic i.e., an onion-skinning fibromuscular proliferation of the intima [29,30]. The anatomic distribution of severity of arteriolosclerosis is important resulting in tissue injury having focal clinical manifestations. Arteriolosclerosis may first affect the arteries of the basal ganglia, then extend into the peripheral white matter, leptomeningeal arteries, and into white matter vessels of the thalamus and cerebellum, and lastly, expands into brainstem arteries [31], [32], [33]. However, not all studies have shown that arteriolosclerosis follows a predictable anatomic sequence [34,35].

There are many modifiable and non-modifiable risk factors that have been described as “upstream” factors that may drive the severity of arteriolosclerosis. Women [36,37] and older black people [24] were more likely to have severe brain arteriolosclerosis than men and whites. APOE4, the major AD risk allele have also been reported to the severity of arteriolosclerosis in people with pathologic AD [38], whereas the ROS and MAP study has shown APOE ε2 has been associated with arteriolosclerosis severity but only among people over 90 years of age at death [39]. Another gene, ABCC9 gene variant (rs704180) was also shown to be a risk factor for arteriolosclerosis severity specifically among persons above 80 years of age at death [36].

Historically, hypertension and diabetes have been considered the most modifiable risk factors for arteriolosclerosis [40,41]. Other epidemiological and animal studies have also reported associations of the severity of arteriolosclerosis with renal failure, sleep fragmentation, obesity, air population, and chemical toxins [36,[42], [43], [44]].

While cross-sectional studies are not ideal to determine upstream vs. downstream factors, some pathologic mechanisms are considered as “downstream” of arteriolosclerosis. Mechanisms associated with arteriolosclerosis include impaired blood flow [45], BBB leakage [46], neurodegenerative mechanisms [47], mechanical stiffening [48], impaired glymphatic system [49], increased oxidative stress [50], neuroinflammation [51], microhemorrhages [52], and microinfarcts [27]. Consistent with this, the community-based ROS and MAP studies indicated persons with severe arteriolosclerosis had an increased burden of microinfarcts, both in cortical and subcortical locations [27]. Moreover, a recent study showed a stronger association between arteriolosclerosis and cortical microinfarcts in people with a higher burden of amyloid-beta, highlighting a possible interaction with neurodegenerative disease [53].

### 2.3 Cerebral amyloid angiopathy

Cerebral amyloid angiopathy (CAA) is a subtype of small vessel disease and is a result of deposition of beta-amyloid in leptomeningeal and parenchymal arteries and arterioles, capillaries, and in rare instances, veins (Fig. 1d). It has been suggested that CAA is the result of an imbalance between Aβ production and Aβ clearance. This is hypothesized to lead to a sequence of destructive changes in the vessel walls, including loss of smooth muscle cells, causing microaneurysms, and fibrinoid necrosis of the vessel wall, resulting in improper blood flow and vessel fragility [54]. CAA is present in the neocortex with more frequent and severe deposition in the occipital region, and later involves vessels in the allocortical regions including the cingulate cortex, hippocampus, entorhinal, and amygdala [55]. CAA can be further classified depending on whether there are amyloid deposits in capillaries i.e. capillary CAA involvement [56,57].

CAA is associated with AD-related risk factors, for example, APOE ε4, rather than traditional vascular risk factors such as hypertension or diabetes [58,59]. Unlike the parenchymal disease, APOE ε2 is also related to an increased burden of CAA though APOE ε4, but not ε2, has been associated with capillary amyloid angiopathy [59]. Additionally, studies also reported two new variants in the UNV5C and C1 gene as non-modifiable genetic risk factors of CAA [60,61].

CAA is common in some but not all pathologic AD cases. Large published clinic-based autopsy series reported that CAA is present in more than 75% of autopsy-confirmed AD brains [62]. However, despite the significant co-occurrence between CAA and AD pathology, CAA remains a clinically and pathologically distinct entity from AD. Not every person with CAA has a pathologic diagnosis of AD, and conversely not all patients with pathologic AD exhibit CAA [63]. In the ROS and MAP studies, 44% of persons with a pathologic AD exhibit moderate-to-severe CAA; by contrast, 20% of persons without pathologic diagnosis of AD had moderate to severe CAA [64]. Pathologically amyloid in AD and CAA is a consequence of abnormal cleavage of amyloid precursor protein (APP) generating a pathogenic amyloid-β protein. However, in contrast to Aβ deposition in AD, which is predominantly composed of Aβ42 variant, Aβ in CAA is composed of the shorter Aβ40 species [65]. Vascular Aβ deposition can lead to vascular degeneration, in particular endothelial cell disruption [66], pericytes degeneration [67], and disruption of smooth muscle [68], leading to decreased vascular compliance and elasticity. CAA-related vascular changes may be responsible for the formation of lobar hemorrhages, microbleeds, cortical microinfarcts, white matter damage, and tissue microstructural changes [69,70]. A study from the Lund Longitudinal Dementia showed a strong association between cortical CAA and cortical microinfarcts [71]. In keeping with this, the ROS and MAP studies further demonstrated a positive strong correlation of moderate-to-severe CAA with the presence of cortical microinfarcts but not with subcortical microinfarcts [27].

### 2.4 Hereditary small vessel disease

Hereditary small vessel disease comprises a group of monogenic disorders which can clinically present as recurrent stroke, vascular dementia, mood conflicts, motor, and cognitive loss, and migraine with visual aura [72]. A small group of disorders identified in this group is cerebral autosomal dominant arteriopathy with subcortical infarcts and leukoencephalopathy (CADASIL), cerebral autosomal recessive arteriopathy with subcortical infarcts and leukoencephalopathy (CARASIL), Fabry disorder, hereditary cerebral hemorrhage with amyloidosis, cerebroretinal vasculopathy, and collagen type IV mutations [72]. Radiology and molecular genetic testing from tissue biopsies, specifically skin biopsies are considered as a diagnostic tool for identifying these disorders.

The most common form of hereditary small vessel disease is CADASIL which is caused by autosomal dominant mutations of the Notch3 gene on chromosome 19p13 that either create or eliminate cysteine residues [73]. The diagnosis of CADASIL is confirmed by the presence of NOTCH3 gene mutations or the presence of granular osmiophilic material seen with electron microscopy in arteriolar media within tissue biopsies [74]. CADASIL patients also appear to demonstrate microvascular changes including apoptosis of smooth muscle cells, dilation of Virchow-Robin spaces, and axonal abnormalities in the frontal white matter [74]. Neuroimaging studies highlighted that most CADASIL patients show extensive white matter hyperintensity lesions located in both subcortical and periventricular white matter regions, cerebral microbleeds, lacunar infarcts in the deep subcortical region (basal ganglia, thalamus) and pons, and brain atrophy [75,76]. Hyperintensities in the external capsule, temporal lobe, and corpus callosum appear to differentiate CADASIL from sporadic small vessel disease [77]. Similar to risk factors of sporadic vessels disease, cardiovascular risk factors such as hypertension, elevated hemoglobin A1c, and smoking are associated with a worse clinical and radiographic phenotype in CADASIL patients [78,79].

Remaining hereditary small-vessel brain disorders are rarer and include CARASIL associated with mutations in the HTRA1 serine peptidase gene on chromosome 10q [72], familial CAA caused by mutations or duplications of the APP β-amyloid precursor protein gene [80], and autosomal dominant retinal vasculopathy with cerebral leukodystrophy caused by frameshift deletions in the exonuclease TREX1 [81]. CARASIL patients present with cognitive dysfunction; they have microvascular changes include reduction of arterial smooth muscle cells and thinning of the arterial adventitial extra cellular matrix resulting in both centrifugally enlarging and collapse of cerebral arteries and finally impaired blood flow [82]. Mutations in type IV collagen subunit genes COL4A1 and COL4A2 have also been reported in association with congenital porencephaly, leukoencephalopathy, or multiple intracranial aneurysms of the carotid siphon [72].

### 2.5 Venous collagenosis

Venous collagenosis (VC) was first summarized by Moody et al. in 1995, characterized as thickening of the lumen wall which results from the deposition of collagen I and III, leading to vessel stenosis or occlusion, most commonly found in the periventricular regions and named as “periventricular VC” [83]. An autopsy study of brains of 24 pathologically confirmed AD and 18 age-matched non-AD patients revealed a potential association between periventricular VC and WMH and suggested potential roles of VC in WMH pathogenesis [84]. Other evidence suggested that VC is associated with pathological changes related to cerebral hypoperfusion, drainage system disruption, and vasogenic edema in the veins around the periventricular white matter, however, further studies are required to understand the contribution and role of VC in VCI and dementia [84,85].

## 3 Neuropathology of vascular-related lesions and tissue injury

### 3.1 Cerebral infarcts

**Fig. 2:**
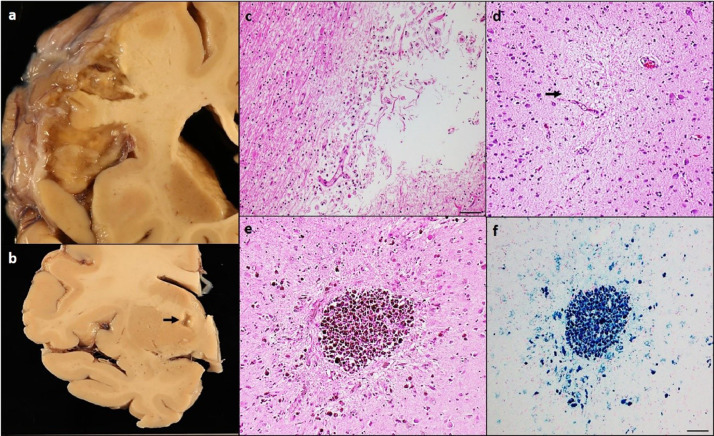
– Pathology of vascular-related tissue injuries in aging brains A chronic macroscopic infarct in the midfrontal cortex (a). Lacunar infarcts in the basal ganglia (arrow, b). Hematoxylin and eosin stain in the mid frontal cortex shows cavitation with hemosiderin-laden macrophages and fibrillary astrocytes (c). Microinfarct in the posterior watershed region (arrow, d). Chronic microhemorrhage in the middle temporal cortex. Routine histology using hematoxylin and eosin stain shows disruption of the vessel wall with accumulation of hemosiderin-laden macrophages (brown deposits, arrow) (e). Perl's iron stain of a chronic microscopic hemorrhage (f). Scale bars: 100µm (image c); 50µm (image d-f).

Cerebral infarcts are important and common cerebrovascular pathology, characterized by the distinct regions of tissue loss identified by the naked eye (macroscopic, Fig. 2a) or under the microscope (microscopic, Fig. 2d) on pathologic studies. Clinic-pathological studies mainly focus on old (chronic) infarcts because cognitive evaluations are often performed months before death and the trajectory of cognitive impairment rather than recent (acute/subacute) infarcts which may be related to perimortem factors.

### 3.2 Macroscopic infarcts

Macroscopic changes in an acute infarct occurring 12-36 h prior to death includes discoloration and slight softening of the brain tissue. An infarct in subacute stages, which occurs within the interval of 2 days to a couple of weeks, is typically associated with cytotoxic edema, resulting in blurring between the demarcation of the white and grey matter. Lastly, a chronic infarct which can occur from an interval of a few months starts to show liquefactive necrosis and cavitation. Chronic macroscopic infarcts are very frequent, occurrence much greater than the frequency of clinical stroke, and present in approximately one-third to one-half of older people [34,86,87].

Pathologically, two different types of macroscopic infarcts that can occur in the aging brain depend on the size. 1. Large brain infarcts (larger than 15mm) are often a result of thrombotic events or thromboembolic occlusion of larger blood vessels. It can occur by a variety of mechanisms such as thrombosis or embolism, with reperfusion in the ischemic infarcted area, and occlusion of arteriolosclerosis- or CAA-affected vessels [88]. 2. Lacunar infarcts are small irregular lesions ranging from 1 to 10mm in size (some include giant lacunes up to 15 mm) most commonly located in the subcortical nuclei (putamen, caudate, globus pallidus, and thalamus), internal capsule, pons, and perhaps less common in the subcortical white matter and cerebellum (Fig. 2b). Significant vascular and/or occlusive changes in smaller penetrating arteries or arterioles (arteriolosclerosis), which branch off from the large arteries in the brain, can lead to decreased perfusion and result in the development of a lacunar infarct.

### 3.3 Microscopic infarcts

Microscopic infarctions are defined as microscopic focal areas of presumably ischemic-related tissue damage frequently associated with astrogliosis and cavitation. Depending upon the interval between onset of ischemic-hypoxic tissue injury and death and similar to macroscopic infarcts, microscopic infarcts can be categorized into acute, subacute, and chronic based on histopathological microscopic appearance. At the onset of infarction, acute lesions typically appear with eosinophilic neurons, pallor, and vacuolization of the tissue. Subacute lesions occurring 3-5 days post-infarct and can be identified by the influx of inflammatory cells, activated macrophages, gemistocytic astrocytes, and rarely neutrophils. Gemistocytic astrocytes develop over the ensuing weeks and over time gradually develop into fibrillary astrocytes as the lesion becomes chronic. Finally, chronic lesion is characterized by cavitation of tissue with fibrillary astrocytes and macrophages, some with hemosiderin-laden [89]. If reperfusion of an embolic infarct occurs, it can often lead to a hemorrhagic infarct [90].

The adult changes in thought (ACT) study showed 16% of the autopsied brain had microinfarcts [91] while data from the ROS study show that a third of subjects (30%) had at least one chronic microinfarct [92]. Multiple studies recognized that microscopic infarcts are commonly associated with macroscopic infarcts but can also be identified in patients with the absence of macroscopic infarcts[12,92]. Indeed, the ROS and MAP studies reported that 45% of persons with microinfarcts did not exhibit macroscopic infarcts [92].

Structural neuroimaging techniques have provided a new avenue to identify microinfarcts but identifying microinfarcts with MRI in vivo remains a challenge in aging. The ultrahigh-field strength of the 7 Tesla (7 T) MRI allows for high-resolution imaging with isotropic voxel sizes to detect “larger” cortical microinfarcts and able to be characterized into cortical or non-cortical microinfarcts [93]. Meanwhile, conventional 1.5 and 3 Tesla are best at detecting infarcts 3 mm or greater in size, therefore most small gross infarcts and microinfarcts are largely invisible to routine neuroimaging [94,95]. Interestingly, Rush ROS and MAP community-based studies were shown that detecting one or more microinfarcts in the brain indicates that there are hundreds of microinfarcts present. For example, obtaining 2 microscopic infarcts in the neuropathologic assessment of the brain is related to an estimated total of 1000 brain microscopic infarcts [96], strengthening the impression that we are only noticing the tip of the iceberg and microscopic vascular lesions are perhaps major contributors to cognitive impairment and it is mostly under-recognized.

### 3.4 Intracranial hemorrhages

Intracranial hemorrhages include large lobar hemorrhages, deep bleeds in the basal ganglia, microscopic brain hemorrhages or cerebral micro-bleeds, and subarachnoid hemorrhages. Microscopic hemorrhages/microbleeds (Fig. 2e and f) typically less than 10 mm in size on neuroimaging are increasingly recognized with imaging techniques. Histologically, microbleeds present with extravasated or lysed erythrocytes indicating an acute stage, whereas the presence of hemosiderin-laden macrophages or presence of iron, with varying degrees of astrocytosis and tissue damage, indicate a chronic stage. ICHs may be the result of a myriad of different vascular and nonvascular factors including hypertensive arteriopathy, cerebral amyloid angiopathy, vascular malformations, or coagulopathies. Older persons with microbleeds often have coexisting white matter hyperintensities (WMHs) and both ischemic and hemorrhagic infarcts.

In aging, CAA is considered a major cause of cortical cerebral hemorrhages, preferring parieto-occipital locations and cortical microbleeds, while arteriolosclerosis or hypertensive vasculopathy is strongly associated with hemorrhages or microbleeds located in hemispheric white matter, subcortical grey matter (basal ganglia and thalamus), brain stem, and cerebellum [97,98]. A recent study reported that the risk of recurrence of intracerebral hemorrhage increases in patients with CAA [99]. While a strong association exists between the presence of CAA and cortical microbleeds, some studies suggest that pathogenic mechanisms other than CAA may be involved in the pathophysiology of cortical microbleeds. A study in the 113 consecutive autopsy series showed no direct topographical association between cortical microbleed and CAA, raising question on the potential pathophysiological mechanism other than CAA and suggesting the possibility of involvement of non-amyloid small vessel disease and other pathogenic mechanisms in the cortical microbleeds pathophysiology [100]. Another study targeting cerebral microbleeds with high-resolution ex vivo 7 T MRI in nine cases with moderate-to-severe CAA and assessed deposition of Aβ in the cortical vessels walls showed low levels of amyloid-β in the microenvironment of cortical microbleeds, reinforcing the possibility of involvement of other mechanisms than CAA damaged vessels in the pathophysiology of microbleeds [101]. Together, these findings validate the need for MRI-histopathology validation studies to bridge the gap between imaging and neuropathology.

### 3.5 White matter lesions

White matter lesions involve pathological changes characterized by white matter rarefaction, demyelination and axonal loss, mild gliosis, edema, and macrophage reaction, often with sparing of subcortical U-fibers [26]. In MRI studies, the term ‘white matter hyperintensities’ is used and characterized as diffuse areas of high signal intensity (hence, “hyperintense”) on T2-weighted or fluid-attenuated inversion recovery sequences [102].

WMHs are more frequent at old age and a few studies report rapid progression with increasing age [103]. Besides aging, female, black population, APOE4 allele presence, and elevated blood pressure have also been associated with WMH progression or burden, however, results are not consistent [103,104]. For instance, in the Cardiovascular Risk Factors, Aging and Incidence of Dementia Study, both midlife and late-life elevated blood pressure were associated with increased WMH risk [105] while another the National Heart Lung and Blood Institute Twin Study identified that only elevated midlife blood pressure was associated to late-life WMH burden [106].

The pathophysiology of white matter histological lesions has been attributed to multiple mechanisms, including defective cerebrovascular reactivity, hypoperfusion, BBB disruption, dysfunction of oligodendrocyte precursor cells, or impaired perivascular (“glymphatic”) clearance [107], [108], [109], [110], [111]. However, knowledge of the underlying pathogenesis of WMH is incomplete and MRI-histopathology correlative studies to cover the degree of WMH-related lesion characteristics are still unclear. Further work is needed to aid the interpretation of WMH in research and clinical setting.

## 4 Clinical pathological correlates of vascular pathologies

The most important cerebrovascular pathology that contributes to the development of VCI is cerebral infarcts. In autopsy studies, cerebral infarcts are defined as discrete regions of tissue loss identified by either naked eye (macroscopic) or under the microscope (microscopic). Because cognitive evaluations are often undergone months before death and recent infarcts including acute and subacute may be related to perimortem factors, clinicopathological studies mainly focus on chronic infarcts.

### 4.1 Macroscopic infarcts

Macroscopic infarcts have long been recognized as a cause of cognitive impairment and a contributor to dementia in aging. In one study, the presence of one or more macroscopic infarctions independently increased the odds of an Alzheimer's type (amnestic) dementia and impaired global cognitive functions and five cognitive domains [10]. Many community-based studies suggest that the larger volumes [11,112] and a higher number of territorial or small subcortical macroscopic infarcts [11,34,[113], [114], [115]] are associated with increased likelihood of dementia and worse cognitive performances. However, there is no clear threshold for determining what volume or number of infarcts require for the development of VCI or dementia. Studies have generally shown an inconsistent relationship between volume/number of infarcts and cognitive impairment. Tomlinson et al described 100 mL of tissue injury required for developing dementia while other shown infarcts with lesser volumes also had dementia [6,112]. These inconsistencies may relate to the location of infarct or the presence of comorbid conditions, such as AD and other neurodegenerative pathologies [116]. Multiple clinic-pathological studies reveal that infarcts in certain brain regions such as the angular gyrus, thalamus, and basal ganglia are more likely to be related to cognitive impairment than other regions [117,118]. However, other studies showed diverse subcortical [117,118] and cortical [11,34,114] infarcts have been related to dementia. Hereby no neuropathological criteria have been currently accepted using macroscopic infarcts to confirm a clinical diagnosis of VCI unlike with Alzheimer's disease and other neurodegenerative diseases.

### 4.2 Microinfarcts

They are recognized as another frequent pathology in aging and even in the absence of macroinfarcts are related to cognitive dysfunction and dementia [92,[119], [120], [121]]. The 90+ population-based study has reported that more than half of persons had microinfarcts, 17% have more than 3 microinfarcts, and microinfarcts present in the cortical regions are associated with dementia [9]. In the ROS study, microinfarcts increased the likelihood of dementia and lower cognition, especially those with multiple cortical microinfarcts. Also, people with the presence of microinfarcts are specifically associated with lower episodic memory, perceptual speed, and semantic memory. In addition, single, multiple, and cortical microinfarcts are associated with worse semantic memory and perceptual speed, but not with episodic memory. Moreover, there was no interaction between microinfarcts and AD or separately with macroscopic infarcts in older individuals to further increase dementia or lower cognitive function [92].

Microinfarcts in the watershed regions are also common, often specified as either anterior watershed regions or posterior watershed regions, location as there is overlapping of all 3 major arterial territories, the anterior, middle, and posterior cerebral arterials. In the event of hemodynamic compromise in the brain (usually in patients with internal carotid stenosis), watershed regions have lower perfusion, creating a hypoxic environment, and producing them more vulnerable to hypoxic-ischemic lesions [122,123]. A recent MAP study defined that persons with multiple watershed microinfarcts had a stronger magnitude of effect on cognitive impairment than the presence of microinfarcts outside the watershed regions [124]. Finally, the precise mechanisms by which these microinfarcts cause cognitive function and dementia are largely not clear, although a few mechanisms including small vessel disease, micro-emboli, cerebral hypoperfusion, or vasoconstriction have been discussed as potential underlying causes [125,126]. In addition, microinfarcts may develop more widespread brain changes such as inflammation, hypoxia, or changes in the BBB that can contribute to dementia. Recent data identified that late-life systolic and diastolic blood pressure as well as a faster decline in systolic blood pressure are associated with an increased number of microinfarcts [127], suggesting another potential indirect mechanism linking blood pressure to microinfarcts.

### 4.3 Hemorrhages

In a similar fashion, various clinic-pathologic studies have linked the presence of intracerebral hemorrhage with cognitive impairment and decline [128], [129], [130]. The mechanisms by which hemorrhages or microbleeds cause cognitive impairment in aging are still debated and may be dependent on the underlying etiology of the hemorrhages. Many studies on cortical microbleeds and dementia show that in persons with cerebrovascular amyloidosis factors include vascular amyloid-β accumulation, activation of vascular injury pathways, and impaired vascular physiology [131], while few studies indicate that microbleeds disrupt structural connectivity and, hence, network function [132,133]. Other studies also demonstrated the disturbance of brain iron metabolism after intracerebral hemorrhage caused by inflammation can also contribute to VCI and dementia [134,135]. Many studies recognized and discuss the clinical significance of the location and numbers of microbleeds present in the brain. For instance, the Rotterdam Scan Study report that patients with microbleeds in cortical or lobar regions predict lower cognitive performance than those who had subcortical microbleeds [136], while a study using data from the Alzheimer's Disease Neuroimaging Initiative (ADNI) shown the presence of high microbleeds count (>4) detected on imaging associated with increased risk of dementia and cognitive deterioration [137]. This study also reported that patients who had lobar microbleeds were associated with a decline in information processing, memory function, and executive function, while microbleeds in other brain regions were associated with decline in motor speed and information processing [137]. It would seem likely that the presence of microbleeds and intracerebral hemorrhage showed the deleterious effect on cognition, but further research is required with careful exclusion of other causes of cognitive impairment to understand the independent contribution of these to VCI.

### 4.4 Vessel disease

There are significant efforts developed to understand the contribution of large/small vessels disease to cognitive impairment and dementia specifically clinical picture judged to be Alzheimer's disease. Atherosclerosis commonly occurs in cerebral arteries and the circle of Willis in the brain in older persons. Several clinic-pathologic epidemiology studies examined specifically intracranial vessels and demonstrated that persons with atherosclerosis pathology had a higher risk of developing dementia [138], [139], [140]. Consistent with others, the ROS and MAP community-based studies suggest that atherosclerosis contributes to the odds of dementia by 30% per level increase in severity and is associated with lower scores in cognitive domains including episodic memory, semantic memory, perceptual speed, and visuospatial abilities. Also, associations remained after taking into account of AD, Lewy body, and gross and microscopic infarcts pathology [35].

Arteriolosclerosis is a common finding at autopsy in older persons and is increased the risk of dementia and cognitive impairment [34,35]. A community-based ROS and MAP study using participants with or without dementia showed that arteriolosclerosis accounted for 33 percent of dementia risk even after controlling for infarcts [35] while a study using the National Alzheimer's Coordinating Center data set suggested severe brain arteriolosclerosis showed worse performances on global cognition in older persons [36]. Moreover, data from over 2000 participants showed the presence of arteriolosclerosis doubles the odds of a poor cognitive trajectory for language when compared to a good trajectory [141].

CAA, another small vessel disease is strongly related to dementia and lowers cognition in older people. The HAAS study identified a relationship between CAA and cognitive impairment [142]. Data from the ROS and MAP studies also indicated the significant role of CAA in cognitive decline in addition to identifying that CAA is independently associated with lowering 3 cognitive domains including episodic memory, semantic memory, and perceptual speed, independent of AD pathology [64]. Moreover, the same study showed that CAA is related to the increased likelihood of dementia even after controlling for other common neuropathologies including AD pathology, Lewy bodies, macroinfarcts, and microinfarcts [64]. However, the potential causative mechanism is not clear to date. As noted previously for CAA related microbleeds, many studies indicate that diffuse brain microbleeds, hypoperfusion, microinfarcts, and white matter hypoxia triggered by vessel changes associated with CAA may be accountable for cognitive decline and dementia, independent of Alzheimer's disease [143,144].

### 4.5 White matter lesion

WML represents as another potential link between vessel disease, cognitive dysfunction, and dementia. There is ample evidence from longitudinal population-based studies that support the cross-sectional association of higher WMH with greater risk of dementia and decrements in global or domain-specific cognitive performance in older persons [102,145,146]. The recent Rush community-based study including participants from the ROS, MAP, and the Minority and Aging Research Study (MARS) has shown that WMHs burden is independently associated with vascular pathologies including arteriolosclerosis and microscopic infarcts in older adults regardless of clinical status [147], volume also was associated with an increased rate of decline in global cognition, perceptual speed, working memory, episodic memory, and semantic memory [148]. Associations between WMH volume and cognitive detriment persisted after adjustment for total gray matter volume, vascular risk factors, and vascular diseases [148]. Importantly, many studies revealed that the clinical relevance of WMH has been largely regulated by their volume and anatomical location in the brain [149]. Overall, current findings from the literature suggest that WMH and white matter changes are complex pathology and have a relationship with dementia and cognition. It is currently unclear whether these additional pathologies represent separate pathological substrates for VCI. Quantitative studies of multiple vascular pathologies in older people with and without dementia with clinical evaluation proximate to death are needed to determine the separate roles of WMHs burden in VCI and other dementias.

### 4.6 Overall contribution of vascular pathology to dementia in aging

A recent study using the ROS and MAP community-based clinic- pathological data estimated the attributable risk for age related amnestic dementia, i.e. clinical presentation judged to be Alzheimer's disease, due to vessels pathologies and neurodegenerative pathologies. Vascular pathologies accounted for over 25% of cases in which about 9% were attributable to macroscopic infarcts, 8% to CAA, 6% to atherosclerosis, and 5% to arteriolosclerosis [150] while attributable risk was 41% for AD pathology, about 11% for Lewy bodies, 5% to hippocampal sclerosis, and about 12% to TDP-43 [150]. The estimated proportion of dementia due to vascular pathologies was somewhat similar with other neuropathologic changes (TDP-43, LBs, and HS) that impacts are considered larger for public health. This data indicates a significant contribution of vascular pathology to cognitive impairment and dementia and shows the area of intense research efforts needed in public health.

## 5 A complex relationship between vascular and neurodegenerative pathologies and dementia

Multiple pathologic studies have reported that most cases of dementia and cognitive impairment have multiple types of pathologies in the brain and raised the possibility that pathophysiologic process of dementia in aging may most often be a cumulative result of neurodegenerative and vascular pathologies. However, understanding the complete spectrum of vascular pathology and the role of vascular pathology with AD and other non-AD neurodegenerative pathologies in VCI remains complex. There are multiple reasons including but not limited to 1) the heterogeneous nature of cerebrovascular lesions, 2) multiple pathogenic mechanisms including changes in cerebral blood flow, neuroinflammation, and disruption of axonal tracts can be related to vascular-related injuries, and 3) there is a lack of a reliable and harmonized diagnostic protocol to assess vascular pathology burden in post-mortem brain tissue that can confirm a clinical diagnosis of VCI. Despite these caveats, there is convincing evidence that vascular pathologies play an important role not only in VCI and VD, but in most of the common age-related dementias including Amnestic dementia. The present section highlights two main questions: Does vascular pathology have a direct or indirect relationship with neurodegenerative pathologies? Does vascular pathology interact with neurodegenerative pathologies to further potentiate dementia? We will explore these questions separately for AD and other non-AD neurodegenerative pathologies.

### 5.1 Relation of vascular pathologies to Alzheimer's disease pathology and dementia

Multiple large longitudinal clinic-neuropathologic studies have reported the co-occurrence of vascular pathology with AD pathology in persons with or without dementia [10,114,116,151], with some showing additive contributions [10,152] or some showing synergistic effects [151,153]to cognitive impairment or dementia. One study showed that one-third of patients with AD present with vascular pathology [154] suggesting that there is a strong vascular component promoting brain injury [87] while findings from the Rush ROS and MAP studies report that about 50% of subjects who have died with AD dementia have mixed pathologies, with the most commonly observed being AD with macroinfarcts [116,155]. Data from oldest-old subjects (over the age of 90 years) of the same cohort study also showed that the prevalence and impact of AD plus infarct pathology including both macroscopic and microscopic infarcts on dementia was greater in the oldest old compared to single pathology [156]. AD mixed with vascular pathology was the most common mixed pathology demonstrated in several studies [114,116,152].

The Nun study specifically showed that participants with a pathologic diagnosis of AD with 1 or multiple subcortical had a higher prevalence of dementia, raisings the possibility of synergistic effects of AD and infarct pathology as well as highlighting the importance of the number and location of lesions [151]. Although subsequent studies also indicated that subcortical infarcts contribute to lowering cognitive function and increasing odds of dementia [10,117,151,153], the effects with AD pathology were additive rather than synergistic [10,153,157,158]. Overall, there is strong evidence that infarcts and AD commonly co-occur, and much, but not all data, suggests an additive rather than synergistic effect with cognition.

Other vascular pathologies, including cerebral amyloid angiopathy, arteriolosclerosis, and atherosclerosis are very common mixed pathologies in aging, especially with AD pathology, and have been shown to contribute to cognitive impairment in late-life even after controlling for AD [35,64,159]. Multiple pathologic studies documented that CAA is commonly associated with AD pathology [64,142,160]. An autopsy study using data from the Rochester epidemiology project reported the occurrence of CAA to be higher in patients with mixed dementia compared to “pure” AD and vascular dementia [160]. An autopsy study from the population-based Honolulu-Asia Aging Study reported the association of CAA with higher mean NFT and NP counts and having at least one APOE-epsilon4, with showing the interaction of CAA with AD pathology to further increase cognitive impairment compared to alone [142]. By contrast, the ROS and MAP studies did not find the interaction between AD and CAA pathology to further the likelihood of dementia or the rate of cognitive decline [64,159]. While recent study using data from the ROS, MAP, and MARS cohort studies of aging highlighted a potential link between neurodegenerative and vascular mechanisms by finding that the association between small vessel disease (both CAA and arteriolosclerosis) and cortical microinfarcts was stronger and more robust in the presence of higher Aβ and tangle burden [53].

Atherosclerosis is another vessel disease that shows the large public health burdens in aging people. There are ample evidences suggesting a relation between atherosclerosis and AD pathology. An autopsy study using 32 AD patients and 22 nondemented controls analyzed atherosclerotic disease by assessing intracranial stenoses based on examining arteries under a dissecting microscope for atheroma-related vessel narrowing and demonstrated that composite measure of the intracranial stenosis burden related with AD neuropathology, including the plaques, tangles, and Braak stage score [161]. Another study using a large dataset of over 1000 subjects from the National Alzheimer's Coordinating Center database established the association of atherosclerosis with increased frequency of neuritic plaques, a major pathologic manifestation of AD with suggesting a role of large vessel disease in AD pathogenesis [162]. Moreover, subsequent studies supported the association between intracranial atherosclerosis and AD after accounting for age, sex, and apolipoprotein E4 allele status [138]. Another study using data from the University of Pennsylvania's Integrated Neurodegenerative Disease Database also confirmed a higher correlation of atherosclerosis severity with multiple metrics of AD pathology including neuritic plaque and paired helical filaments tau neurofibrillary tangle. The authors suggest that atherosclerosis and Alzheimer's disease are interrelated and that common aetiologic or reciprocally synergistic pathophysiological mechanisms promote both vascular pathology and plaque and tangle pathology [25]. Meanwhile, some studies fail to show an association of cerebrovascular atherosclerosis with AD pathology [163], [164], [165].

Arteriosclerosis has been variably associated with AD pathology. Some clinical studies that have focused on identifying the relation of arteriolosclerosis with AD pathology, have not detected an association [35,166]. One study established evidence of hypoxia-linked gene expression in the AD patient's brains [167], yet, the effect was not related to arteriolosclerosis severity. Meanwhile a recent study from the ROS and MAP cohorts indicate an interaction between arteriolosclerosis and amyloid-beta burden to increase the odds of microinfarcts in the brain [53]. This observation suggests a link between AD pathology and vessel pathologies in the development of tissue injury, i.e. microinfarcts, that cause exacerbate cognitive impairment and lower the threshold for dementia. Further studies examining the relationship or interactions between arteriolosclerosis and other pathologic changes specific to AD such as synaptic loss, tau, Aβ-related pathologic changes are warranted to understand the mechanistic link between AD and vascular pathology in the development of cognitive changes.

Overall, these studies suggest that vessel pathologies mixed with AD pathology are very common in the brain of older people. There is intriguing literature suggesting a synergistic effect between vessel and AD pathologies, both on tissue injury and cognitive impairment. Meanwhile, most literature shows that vessel pathologies directly add to the likelihood of dementia or VCI. The contribution of vessel pathologies to AD pathology and mechanisms in VCI in mixed vascular and AD dementia remains an area of intense research efforts. Common vascular risk factors, interactions, and mechanisms of injury from AD and vascular pathologies also remain topics of great interest.

### 5.2 Relation of vascular pathologies to non-AD neurodegenerative pathologies

There are other non-AD neurodegenerative pathologies such as limbic-predominant age-related TDP-43 encephalopathy-neuropathologic change (LATE-NC) that commonly exist in the brains of older people and co-occur with AD pathology [168]. The role of vascular pathology is likely to be a growing health problem in people with another neurodegenerative diseases since other non-AD neurodegenerative and vascular pathologies continue to increase in aging brains. While there are very limited studies carried out to examine the relationship between vascular pathologies and other non-AD neurodegenerative pathologies.

Recently, LATE-NC concept has been described as a common TDP-43 proteinopathy which initially manifests in the amygdala and then extends to other limbic (hippocampus and entorhinal cortex), and cortical regions, present in 15-20 percent of clinically diagnosed AD autopsy cases, associated with an accelerated rate of cognitive decline and produces AD-like clinical symptoms [168]. Few studies have focused on the occurrence of LATE-NC in people with small vessels disease and shown that LATE-NC is more frequent in persons with moderate-to-severe arteriolosclerosis than in those with none/low arteriolosclerosis severity [169], [170], [171]. Recent pathologic data from the ROS, MAP, and MARS studies examined the co-occurrence and association of LATE-NC with microvascular pathologies including arteriolosclerosis in three brain regions (basal ganglia, anterior watershed, and posterior watershed), CAA, microinfarcts and reported that 85% of persons with LATE-NC had one or more microvascular pathologies with suggesting that LATE-NC with microvascular pathology is a very common mixed pathology [172]. Also, the same study strongly reported that the presence of capillary CAA and severity of arteriolosclerosis in the posterior watershed region was related with higher odds of LATE-NC stage, with suggesting that small vessel disease pathology may contribute to LATE-NC in older people [172]. These findings were consistent with two more recent studies that further strengthened the link between cerebrovascular defects and TDP-43. First, Bourassa et al. indicated reduced brain arteriolar mural cell markers (α-SMA for smooth muscle cells, PDGFRβ and CD13/ aminopeptidase N for pericytes) in the parietal cortex in brains with comorbid LATE-NC [173]. Second, Belvins et al. indicated a prevalence of TDP-43 deposits outside both capillaries and arterioles [40].

HS pathology is increasingly recognized in the aging brain particularly the oldest old (90+) and refers to the pathology of severe neuronal loss and gliosis of CA1 and /or subiculum. TDP-43 proteinopathy is associated with increased HS pathology [174,175]. Cerebral ischemia is considered an etiological mechanism that may play a major role in the pathogenesis of HS [118]. This phenomenon was supported by a study using data from multiple large autopsy series to investigate the association between cerebrovascular pathology and HS aging and showed a strong association of arteriolosclerosis in the frontal cortex with HS [166]. In contrast, the ROS, MAP and MARS studies failed to report the association between vessels pathology and HS, but further research is warranted with a larger sample size [172].

Overall, these findings raise questions on how LATE-NC/HS and vascular pathology particularly arteriolosclerosis and capillary CAA interact mechanistically. There is a critical question that remains open as to which one acts as upstream and which plays as downstream in dementia disorder. It could be possible that small vessel disease may contribute to increased non-AD neurodegenerative pathology or vice versa, or there may be a synergistic feed-forward reaction in which each pathology exacerbates the other to affect neurologic function.

## 6 Pathophysiologic mechanism contributing to VCI

### 6.1 Importance of the neurovascular unit

The neurovascular unit (NVU) is known to generate a protecting biochemical barrier between the cerebral microenvironment and the peripheral circulation, composed of glial cells (astrocytes, microglia, oligodendrocytes), neurons, endothelial cells, pericytes, basal membrane, and extracellular matrix [176]. The components of NVU play a crucial role in regulating the cerebral blood flow (CBF) and maintaining the blood-brain barrier (BBB), and contribute to immune surveillance [6,177]. Because NVU is a vital structure in maintaining cerebral homeostasis, the dysfunction of its components can be involved in the pathogenesis of VCI. There are common morphofunctional changes that occur during pathological processes such as oxidative stress, inflammation, degradation of the extracellular matrix and basal lamina components, and loss of selectivity of the BBB. A study showed that pericyte degeneration due to ischemia contributes to cerebral homeostasis failure [178].

### 6.2 Endothelial dysfunction

The brain requires cerebral blood flow with a local metabolic rate to provide essential distribution of nutrients and oxygen in the brain. The proper cerebral blood flow is the key element in neuronal functioning therefore, disruption of CBF can lead to brain dysfunction and death [179]. To maintain stable CBF, cerebral blood vessels have an inherent capability to keep CBF relatively constant across the range of blood pressures. This hemodynamic process is known as cerebral autoregulation that defends the brain from unwanted swings in arterial pressure associated with daily living activities and provides a stable CBF baseline [180]

Studies have reported a strong link between cerebral endothelial dysfunction, CBF reduction, and VCI development [181]. Under physiological conditions, receptors on the endothelial cells release potent signaling molecules such as nitric oxide, prostanoids, and endothelin that take part in the coordination of vasodilatation/vasoconstriction and CBF regulation [182]. Therefore, it is believed that endothelial dysfunction causes BBB compromise, exposes neural cells to harmful substances, affects neurovascular coupling, and making CBF cannot respond timely to neuronal activity and contribute to hemostatic imbalance [182], [183], [184]. In addition, cerebral endothelial cells are connected by tight junctions and coupled with specialized transport proteins that regulate the trafficking of hydrophilic substances between the brain and blood, a key feature of the BBB [185]. For example, amino acid and GLUT1transporters regulate the transmission of amino acids and glucose, respectively into the brain, whereas LRP-1, ABC transporters, and others, eliminate drugs and metabolic by-products from the brain, including Aβ and lactate [186]. Despite this, the mechanism underlying the endothelial cells dysfunction has not been completely elucidated. Patients with VCI often seem with focal changes in cerebral endothelial cells, including increased pinocytosis, decreased mitochondrial content, loss of tight junctions, decreased capillary density, and disruption of BBB [187], [188], [189]. These changes might have occurred before the onset of VCI thereby procedures that can improve cerebral endothelial cells function could slow down or prevent VCI.

In addition, endothelial cells support neuronal survival and protect them from injury [190], promotes the proliferation and survival of oligodendrocytes [191], interact with astrocytes for maintenance of BBB [192]. Therefore, neurovascular cells are metabolically and tropically interdependent in such a way that damage to one cell type eliminates a vital source of support to the whole unit and produce deleterious consequences for the other cell types.

### 6.3 Oxidative stress and inflammation

Oxidative stress and inflammation are currently thought to be key pathogenic factors in the development of neurovascular dysfunction, VCI, and dementia [193,194]. Several experimental studies indicate that reactive oxygen species stimulated by NADPH oxidase are responsible for the cerebrovascular changes induced by VCI risk factors such as hypertension, insulin resistance, and diabetes [195], [196], [197]. For instance, patients with hypertension exhibit dysfunction in autoregulation of cerebral perfusion in response to changes in systemic blood pressure. Experimental studies revealed that hypertension leads to activation of abnormal signaling of angiotensin II (vasoactive peptide), regulate remodeling of blood vessels in response to blood-pressure dysregulation that finally promotes pro-inflammatory effects through activation of leukocytes, cell adhesion molecules, and inflammatory cytokines as well as the stimulation of NADPH oxidase, which has emerged as an important source in vascular oxidative stress and reactive oxygen species [194,198]. Generation of reactive oxygen species by oxidative stress can also induce endothelial dysfunction and release vascular endothelial growth factors and prostanoids that promote vascular leakage, protein extravasation, and cytokine production [199]. In addition, several studies report that NADPH oxidase-induced reactive oxygen species can initiate and promote inflammatory pathways through Toll-like receptors. Inflammation, in turn, enhances oxidative stress by increasing the expression of reactive oxygen species–producing enzymes and down-regulating the expression of enzymes responsible for antioxidant defenses [200].

Pathological studies have shown upregulated expression of oxidative stress and inflammation marker, including cytokines, microglial activation, reactive astrocytes, isoprostanes in the damaged white matter associated with VCI [201], [202], [203]. The mechanisms of these responses have not been fully elucidated, but animal studies indicate that cerebral hypoperfusion is associated with white matter inflammation and oxidative stress [204], [205], [206], indicating that hypoxia-ischemia might be one of the factors to trigger these responses. In addition, BBB disruption is also important to activate additional oxidative stress by inducing tissue hypoxia and by extravasation of plasma proteins, such as fibrinogen, immunoglobins, complement, and Aβ, which enter into brain tissue, serve as potential activators of inflammation and production of free radicals [207].

In addition, studies indicate that the downregulation of low-density lipoprotein receptor-related protein-1, a critical Aβ brain clearance receptor in cerebral blood vessels of patients with Alzheimer's disease, leads to accumulation of amyloid around blood vessels and worsening of vascular dysfunction [208]. Furthermore, oxidative stress reduces the production of brain-derived neurotrophic factors by the endothelium of vessels [190] and contributes to brain atrophy associated with VCI [209].

## 7 Overview of cerebrovascular pathologies: update from the religious orders study and rush memory and aging project

**Fig. 3:**
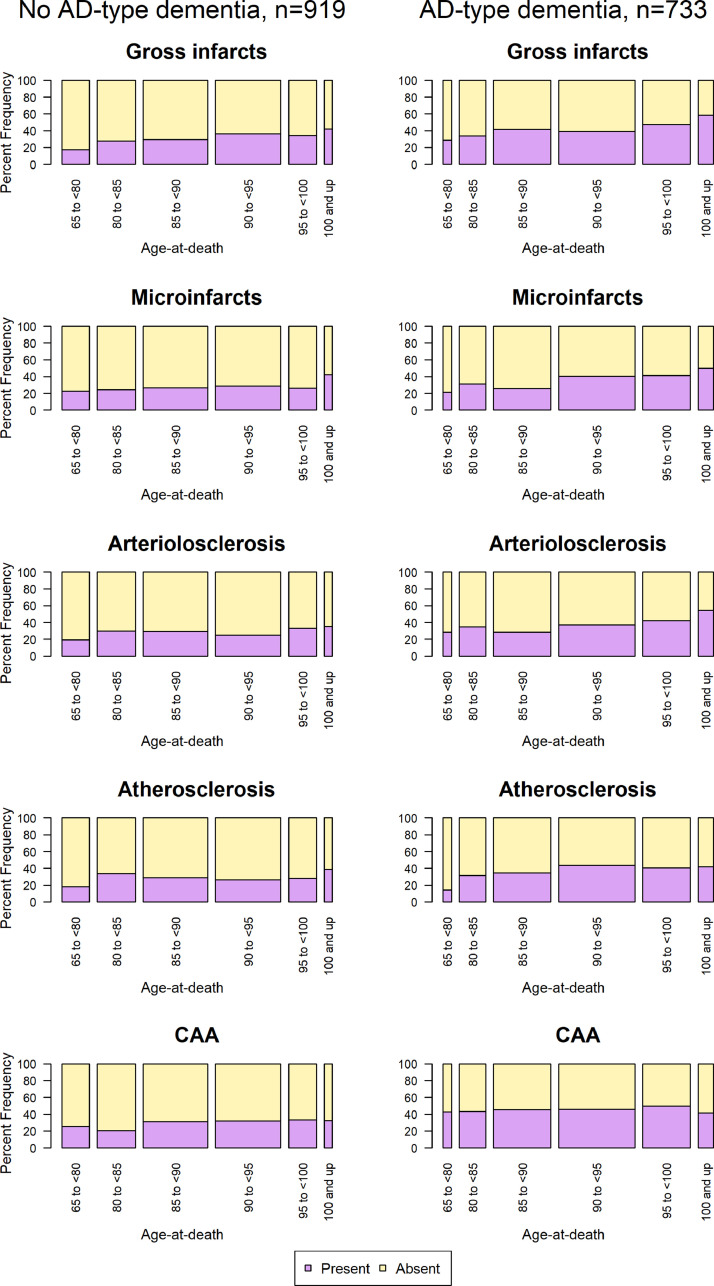
CVD pathologies in ROS and MAP subjects. Frequency of vascular pathologies (gross infarcts, microinfarcts, arteriolosclerosis, atherosclerosis, and CAA) in the ROS and MAP subjects (with no-AD type dementia and with AD-type dementia) with increased age described in Table 1.

Here we provide an update on the frequency of vascular pathologies collected from the ROS and MAP studies in people with and without dementia that represented to be Alzheimer's disease across ages. We included 1652 consecutive autopsied subjects (mean age of death, 89.3 years, SD = 6.66; 67% women; mean education, 16.28 years, SD = 3.63) that have complete diagnostic data of dementia and pathologic assessments for chronic macroscopic and microscopic infarcts, moderate-to-severe atherosclerosis, arteriolosclerosis, and CAA, and AD-neuropathologic change (NIA-Reagan criteria intermediate or high). Each of these pathologies has been previously shown to lower the threshold for cognitive impairment and contribute to dementia. Fig. 3 and Table 1 show the frequency of occurrence of vascular pathologies in people with dementia (probable and possible) and without dementia (normal cognitive impairment and mild cognitive impairment people) with every 5-15 additional years of age (age 65-<80 years, 80-<85 years, 85-<90 years, 90-<95 years, 95-<100 years, and ≥100 years). As previously described, we found that vascular pathologies are common in older persons who die from dementia, with each separate pathology observed in more than one-third of those with dementia, and about one-fourth in older persons with no dementia proximate to death. Results also depict how vessel pathologies evolve with aging in both diagnostic groups. First, the occurrence of vessels pathologies, particularly arteriolosclerosis, and cerebral infarcts (both macroscopic and microscopic) appear to increase with advancing age. Indeed, among centenarians with dementia, arteriolosclerosis, macroscopic, and microscopic infarcts were each present in about half or more of the subjects. Second, the frequency of vascular pathology in those without dementia, though lower than those with dementia, also continues to rise with age, suggesting a lower burden of vascular pathology in this group or better reserve mechanisms.

**Table 1:** Frequency of CVD pathology in participants with and without AD-type dementia with increase age.

Diagnostic groups	CVD Pathology	Age-at-death						p-value
		65-<80 yrs (*n* = 110)	80-<85 yrs (*n* = 152)	85-<90 yrs (*n* = 256)	90-<95 yrs (*n* = 259)	95-<100 yrs (*n* = 111)	≥100 yrs (*n* = 31)	
People without AD-type dementia (*n* = 919)	Gross infarcts (*n* = 281, 31%)	19 (17.27)	42 (27.63)	75 (29.30)	94 (36.29)	38 (34.23)	13 (41.94)	X^2^=16.56, DF=5, p= 0.005
	Microinfarcts (*n* = 246, 27%)	25 (22.73)	37 (24.34)	68 (26.56)	74 (28.57)	29 (26.13)	13 (41.94)	X^2^=5.46, DF=5, p=0.361
	Arteriolosclerosis (*n* = 253, 28%)	21 (19.09)	45 (29.61)	75 (29.30)	64 (24.71)	37 (33.33)	11 (35.48)	X^2^=8.54, DF=5, p=0.128
	Atherosclerosis (*n* = 279, 28%)	20 (18.18)	51 (33.55)	74 (28.91)	68 (26.25)	31 (27.93)	12 (38.71)	X^2^=9.86, DF=5, p=0.079
	CAA (*n* = 269, 29%)	28 (25.45)	31 (20.39)	80 (31.25)	83 (32.05)	37 (33.33)	10 (32.26)	X^2^=9.02, DF=5, p=0.108
People with AD-type dementia (*n* = 733)		65-<80 yrs (*n* = 28)	80-<85 yrs (*n* = 83)	85-<90 yrs (*n* = 182)	90-<95 yrs (*n* = 241)	95-<100 yrs (*n* = 151)	≥100 yrs (*n* = 48)	
	Gross infarcts (*n* = 305, 42%)	8 (28.57)	28 (33.73)	76 (41.76)	94 (39)	71 (47.02)	28 (58.33)	X^2^=12.09, DF=5, p=0.033
	Microinfarcts (*n* = 262, 36%)	6 (21.43)	26 (31.33)	47 (25.82)	97 (40.25)	62 (41.06)	24 (50.00)	X^2^=19.23, DF=5, p=0.001
	Arteriolosclerosis (*n* = 268, 37%)	8 (28.57)	29 (34.94)	52 (28.57)	89 (36.93)	64 (42.38)	26 (54.17)	X^2^=14.50, DF=5, p=0.012
	Atherosclerosis (*n* = 279, 38%)	4 (14.29)	26 (31.33)	63 (34.62)	105 (43.57)	61 (40.40)	20 (41.67)	X^2^=12.94, DF=5, p=0.023
	CAA (*n* = 337, 46%)	12 (42.86)	36 (43.37)	83 (45.60)	111 (46.06)	75 (49.67)	20 (41.67)	X^2^=1.53, DF=5, p=0.909

**Fig. 4:**
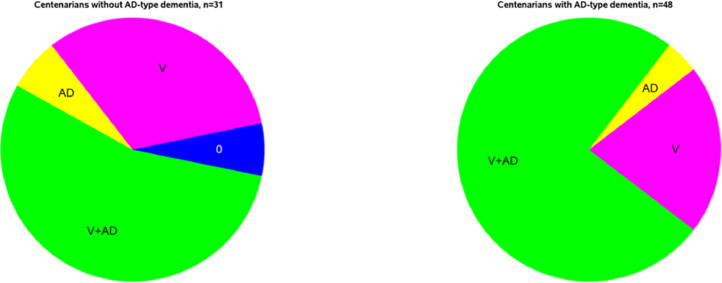
Mixed AD and vascular pathologies in the ROS and MAP Centenarians. Frequency of mixed pathologies in ROS and MAP centenarians with no-AD type dementia and with AD-type dementia described in Table 2. V = vascular without AD, AD = Alzheimer's Disease without vascular, 0 = no vascular or AD pathology.

In Table 2 we show the frequency of a pathologic diagnosis of AD alone, significant vascular pathology alone, and mixed AD pathologic diagnosis and vascular pathology in persons with and without dementia across ages. Many striking aspects are note-worthy. First, the frequency of AD mixed with any vascular pathology is over 50% in even the younger age groups with dementia with the overall frequency of mixed AD and vascular pathology, not surprisingly, rising across age. Second, vascular pathology in the absence of a pathologic diagnosis of AD is also high across all age groups with a diagnosis of dementia (in community setting without biomarkers). Third, though the number of centenarians in the cohort with dementia was relatively small, almost all (>95%) had significant vascular pathology. Indeed, the vast majority of centenarians with AD pathology, have evidence of AD mixed with vascular pathology, and only very rarely AD without vascular pathology exist (Fig. 4). Finally, it is important to note that 21% of centenarians with a community diagnosis of dementia had vascular pathology without the presence of AD, which likely indicates evidence of vascular dementia, though the role of other neurodegenerative pathologies such as LBs and LATE-NC were not examined in this study. The current understanding of clinico-pathological correlation with dementia in centenarians is extremely limited [210], [211], [212]. Future study with a specific focus on identifying pathology that is associated with dementia and factors contributing to the prevention or delay of age-related cognitive decline in centenarians is required to understand the complexity of dementia in the oldest old.

**Table 2:** Frequency of CVD and mixed AD pathology in participants with and without AD-type dementia across ages.

Diagnostic groups	Pathology	Age-at-death					
		65-<80 yrs (*n* = 110)	80-<85 yrs (*n* = 152)	85-<90 yrs (*n* = 256)	90-<95 yrs (*n* = 259)	95-<100 yrs (*n* = 111)	≥100 yrs (*n* = 31)
Without AD-type dementia (*n* = 919)	No vascular and AD (*n* = 125)	28 (25.45)	26 (17.11)	36 (14.06)	26 (10.04)	7 (6.31)	2 (6.45)
	Vascular only (*n* = 327)	51 (46.36)	65 (42.76)	83 (32.42)	86 (33.20)	32 (28.83)	10 (32.26)
	AD only (*n* = 100)	9 (8.18)	23 (15.13)	21 (8.20)	29 (11.2)	16 (14.41)	2 (6.45)
	Vascular + AD (*n* = 367)	22 (20)	38 (25)	116 (45.31)	118 (45.56)	56 (50.45)	17 (54.84)
With AD-type dementia (*n* = 733)		65-<80 yrs (*n* = 28)	80-<85 yrs (*n* = 83)	85-<90 yrs (*n* = 182)	90-<95 yrs (*n* = 241)	95-<100 yrs (*n* = 151)	≥100 yrs (*n* = 48)
	No vascular and AD (*n* = 19)	2 (7.14)	4 (4.82)	5 (2.75)	6 (2.49)	2 (1.32)	0 (0.00)
	Vascular only (*n* = 105)	5 (17.85)	12 (14.46)	28 (15.38)	30 (12.45)	20 (13.25)	10 (20.83)
	AD only (*n* = 75)	4 (14.29)	15 (18.07)	20 (10.99)	23 (9.54)	11 (7.28)	2 (4.17)
	Vascular + AD (*n* = 534)	17 (60.71)	52 (62.65)	129 (70.88)	182 (75.52)	118 (78.15)	36 (75)

## 8 Summary and future directions

There is variability, heterogeneity, and complexity in the vascular pathologies that lead to the progression of VCI and dementia and that contribute to cognitive decline. Certainly, it is clear that vascular pathologies most commonly coexist in the brains of older persons with AD pathology, increasing the risk and severity of cognitive impairment. Because of the high incidence of mixed pathologies, it is important to investigate common risk factors such as APOE isoforms and pathogenic mechanisms such as neuroinflammation and oxidative stress which underlie multiple pathologies. While there have been strong advances in imaging biomarkers to identify microbleeds, large infarcts, and white matter changes in-vivo, specific structural biomarkers are necessary to identify other pathologies including small vessels disease and microinfarcts further understand and aid the diagnosis of VCI. Indeed, in the neuropathology field, there is an increasing need to validate and standardize an approach to evaluate vascular pathology in post-mortem brain tissue. However, the use of ex-vivo imaging techniques has continued to complement post-mortem neuropathologic evaluations. More studies are needed to fully understand the relationship between vascular biology, vasculature, and vascular tissue injuries with cognitive outcomes. Indeed, we are not capturing the whole picture in relation to cognitive decline, and there is likely much more unrecognized vascular pathology that may play important roles in VCI and explaining the variance in cognitive decline in aging.

## Declaration of Competing Interest

There are no conflicts of interest.
